# Penta-1,2,3,4,6-*O*-Galloyl-β-d-Glucose Inhibits UVB-Induced Photoaging by Targeting PAK1 and JNK1

**DOI:** 10.3390/antiox8110561

**Published:** 2019-11-15

**Authors:** Ji-An Kim, Jae-Eun Lee, Ji Hye Kim, Hyo-Jeong Lee, Nam Joo Kang

**Affiliations:** 1 School of Food Science and Biotechnology, Kyungpook National University, Daegu 41566, Korea; kdy4401@naver.com (J.-A.K.); lju1033@naver.com (J.-E.L.); friend8201@naver.com (J.H.K.); 2 Korean Medicine (KM)-Application Center, Korea Institute of Oriental Medicine (KIOM), Daegu 41062, Korea; 3 College of Korean Medicine, Cancer Preventive Material Research Center, Kyunghee University, Seoul 02447, Korea; strong79@khu.ac.kr

**Keywords:** penta-1,2,3,4,6-*O*-galloyl-β-d-glucose, JNK1, PAK1, photoaging, ultraviolet B

## Abstract

Penta-*O*-galloyl-β-d-glucose (PGG) is a gallotannin polyphenolic compound that occurs naturally in fermented *Rhus verniciflua*. The present study aimed to examine the effect of PGG on UVB-induced skin aging and its molecular mechanisms in HaCaT human keratinocytes and SKH-1 hairless mice models. PGG suppressed UVB-induced matrix metalloproteinase-1 (MMP-1) expression in HaCaT cells by inhibiting phosphorylation of RAF/MEK/ERK, MKK3/6/p38, and c-Jun. UVB-induced ERK and p38 signaling pathways that induce the MMP-1 expression were mediated by PAK1 in HaCaT cells. PGG suppressed PAK1 and JNK1 kinase activities, and directly bound both PAK1 in an ATP-competitive manner and JNK1 in an ATP-noncompetitive manner. Consistently, PGG decreased UVB-induced wrinkle formation, epidermal thickness, type 1 collagen and MMP-13 expression in mouse skin. Overall, these results indicate that PGG exhibits anti-photoaging effects in vitro and in vivo by the suppression of PAK1 and JNK1 kinase activities, and may be useful for the prevention of skin aging.

## 1. Introduction

The skin, unlike other organs, is directly influenced by the environment and therefore undergoes skin aging following repeated exposure to environmental stresses including ultraviolet (UV) light [1,2]. Sun-damaged skin is characterized by increased wrinkling, sagging and pigmentary changes [2]. UV light can be divided into three types: UVA, UVB and UVC. UVB can markedly induce biological damage on the skin and significantly affects the epidermis, causing premature skin aging [3,4].

Matrix metalloproteinases (MMPs) are a large family of zinc-dependent endopeptidases with a broad substrate specificity [1,5]. MMPs play important roles in angiogenesis, tumor invasion and photoaging [6]. In particular, MMP-1 is the leading member of the interstitial collagenase subfamily of MMPs [7]. MMP-1 is also associated with damage to connective tissues, such as type 1 and type 3 collagens, found in photo-exposed skin [8]. Therefore, MMP-1 is considered a major marker of skin aging, and suppressing MMP-1 expression or activity has been considered a good strategy for preventing wrinkle formation and photoaging [6,9,10].

UV activates several growth factor receptors or cytokines on the cell surface and subsequently activates proteins belonging to the MAPK family, including extracellular signal regulated kinases (ERKs), c-Jun N-terminal kinases (JNKs), and p38, by phosphorylation [11,12].

These activated MAPKs play important roles in signaling pathways that regulate the expression of several MMPs, including MMP-1. p21-activated kinase (PAK), a family of serine/threonine kinases conserved from yeast to human, plays a regulatory role in cytoskeleton, cell migration and cell cycle progression [13,14]. PAK1 contributes to the activation of signaling pathways involving MAPKs, and can be induced by cross-talk with the MAPK signaling pathway in epithelial cells under a variety of conditions, including several stimulations [15,16,17]. However, the involvement of PAK1 in UVB-induced skin aging remains unclear.

**Figure 1:**
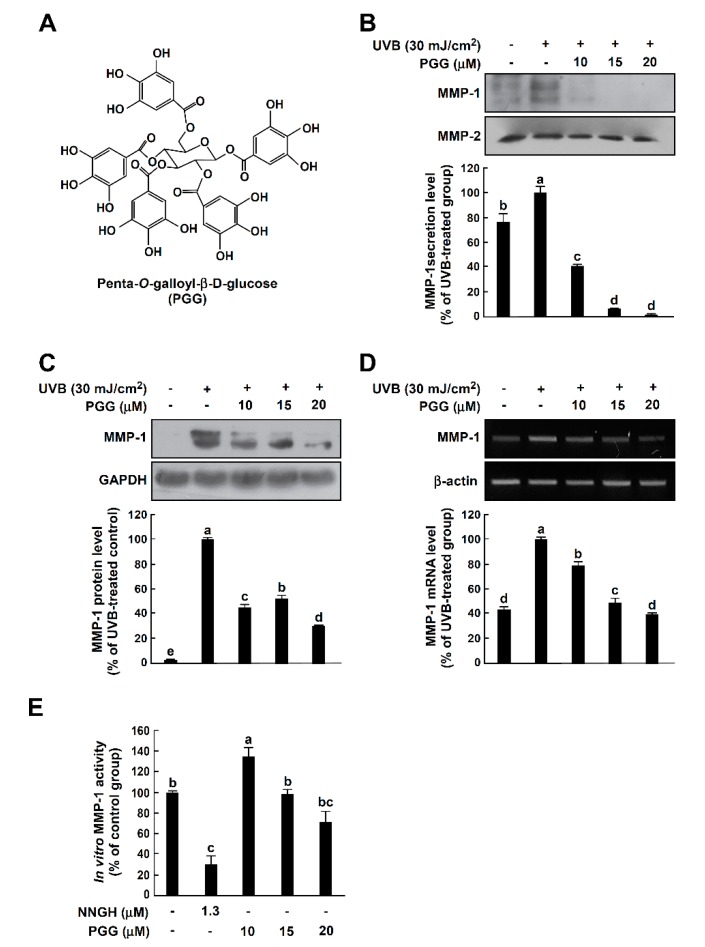
Effect of PGG on UVB-induced MMP-1 expression in HaCaT cells. (**A**) Chemical structure of PGG. (**B**) Effect of PGG on UVB-induced MMP-1 secretion in cell culture media. PGG (10, 15 and 20 μM) was treated for 1 h before UVB exposure (30 mJ/cm^2^) to cells. After 48 h, conditioned culture media were collected. Western blot analysis using specific antibodies against MMP-1 protein was performed to determine levels of MMP-1 secretion. (**C**) Effect of PGG on UVB-induced MMP-1 expression in HaCaT cells. Cells were treated with PGG (10, 15 and 20 μM) for 1 h before being exposed to UVB (30 mJ/cm^2^) and harvested 6 h later. Western blot analysis using specific antibodies against MMP-1 protein was performed to determine MMP-1 expression levels. GAPDH was used as loading control. (**D**) Effect of PGG on UVB-induced MMP-1 mRNA level in HaCaT cells. Cells were treated with PGG (10, 15 and 20 μM) for 1 h before being exposed to UVB (30 mJ/cm^2^) and harvested 4 h later. MMP-1 mRNA level was determined by reverse transcription (RT)-PCR. Data are representative of three independent experiments that yielded similar results. (**E**) Inhibitory effect of PGG on in vitro MMP-1 activity assay. MMP-1 activity is expressed as percentage of relative activity to untreated control MMP-1 activity. Data are presented as the mean ± standard deviation (S.D.), as determined from three independent experiments. Values that do not share common letter (a,b,c,d,e) on bar indicate statistically significant difference from each other (*p* < 0.05).

Penta-1,2,3,4,6-*O*-galloyl-β-d-glucose (PGG, Figure 1A) is a gallotannin polyphenolic compound that occurs naturally in fermented *Rhus verniciflua* [18,19]. Several in vitro studies have reported multiple biological activities, including potential chemopreventive effects, in prostate cancer cells and other cancer cell lines [20,21,22,23]. Moreover, it was recently reported that PGG significantly induced type II collagen expression in rabbit articular chondrocytes [24].

Recently, PGG has been reported to possess photoprotective capacity by targeting NF-κB and MAPK signaling in UVB-induced human dermal fibroblasts and mouse skin [25]. Based on the above studies, PGG could represent an active pharmaceutical and cosmetic agent. However, the preventive effect on photoaging and molecular mechanisms of PGG have not been fully understood.

In the present study, we examined the effect of PGG on UVB-induced skin aging using HaCaT human keratinocytes and SKH-1 hairless mice. We found that PGG suppressed UVB-induced secretion and expression of MMP-1 in HaCaT cells by targeting PAK1 and JNK1, and attenuated UVB-induced photoaging in vivo.

## 2. Materials and Methods

### 2.1. Chemicals and Antibodies

PGG and fetal bovine serum (FBS) and an antibody against β-actin were obtained from Sigma-Aldrich (St. Louis, MO, USA). Dulbecco’s Modified Eagle’s Medium (DMEM), penicillin-streptomycin and 0.25% trypsin-EDTA were purchased from GIBCO^®^ Invitrogen (Auckland, New Zealand). Anti-human MMP-1 antibody was purchased from Neomarker (Fremont, CA, USA). Antibodies against human MMP-2, phosphorylated c-RAF (Ser338), MEK1/2 (Ser217/221), JNK (Thr183/Tyr185), MKK4 (Ser257/Thr261), MKK3/6 (Ser189/207), and PAK1 (Ser144), and total c-RAF, MEK1/2, MKK4, MKK3/6, and PAK1 were purchased from Cell Signaling Biotechnology (Danvers, MA, USA). An antibody against phosphorylated p38 (Thr180/Tyr182) was purchased from BD Bioscience (San Jose, CA, USA). Antibodies against phosphorylated ERK1/2 (Thr202/Tyr 204), total ERK1/2, total p38, and GAPDH were obtained from Santa Cruz Biotechnology (Santa Cruz, CA, USA). p21-activated kinase inhibitor III (IPA-3) was purchased from Millipore (Billerica, MA, USA). Recombinant active JNK1 protein was obtained from Upstate Biotechnology (Lake Placid, NY, USA). MMP-1 Fluorometric Drug Discovery Kit was purchased from Enzo Life Science (Farmingdale, NY, USA). The ADP-Glo^TM^ Kinase Assay Kit and recombinant active PAK1 protein were obtained from Promega (Fitchburg, WI, USA). Protein Assay Kit was purchased from Bio-Rad Laboratories (Hercules, CA, USA).

### 2.2. Cell Culture and UVB Irradiation

HaCaT human keratinocytes were cultured in DMEM supplemented with 10% FBS and 1% penicillin/streptomycin at 37 °C in humidified atmosphere with 5% CO_2_. UVB irradiation was performed using a Bio-Link Cross linker (Vilber Lourmat, Torcy, France), emitting wavelengths with peak emission at 312 nm. HaCaT cells were starved for 24 h in serum-free DMEM and treated with PGG (10, 15 or 20 μM) prior to UVB exposure. After 1 h, cells were washed with PBS and irradiated with UVB (30 mJ/cm^2^) in a small volume of PBS.

### 2.3. In Vitro MMP-1 Activity Assay

The inhibitory effect of PGG on MMP-1 enzymatic activity was analyzed using a MMP-1 Fluorometric Drug Discovery Kit (Enzo Life Sciences) following the manufacturer’s instructions. The kit included a broad inhibitor NNGH, which served as a control. The mixture containing human recombinant MMP-1 was incubated at 37 °C for 30 to 60 min. MMP-1 activity was measured using substrate, and the fluorescent signal was detected at Ex/Em = 540/590 nm. MMP-1 activity was calculated as follows: MMP-1 activity/(ABU of sample-ABU of MMP control). Samples were compared to a control that contained assay buffer instead of sample (100% of MMP activity).

### 2.4. MTT Assay

2 × 10^3^ cells/well of HaCaT cell were seeded into 96-well cell culture plates and incubated at 37 °C. After 24 h, the attached HaCaT cells were treated with PGG at indicated concentrations. The cells were further incubated with PGG for 24 h, and then treated with MTT solution to a final concentration of 1 mg/mL for 2 h. The culture media were removed and 200 μL/well of DMSO was treated to dissolve MTT formazan crystal. The dissolved MTT formazan crystal was measured by absorbance at 570 nm using a microplate reader (Sunrise-Basic, Tecan, Groding, Austria).

### 2.5. Western Blot Analysis

Cells (1 × 10^5^) were cultured in a 60-mm diameter dish for 24 h and then starved in serum-free medium for an additional 24 h to eliminate the influence of FBS on kinase activation. Cells were then treated with PGG (10, 15 or 20 µM) for 1 h prior to UVB exposure (30 mJ/cm^2^) for the indicated periods. To determine the amount of secreted MMP-1 into culture media, equal aliquots of conditioned culture media from an equal number of cells were fractionated by 10% sodium dodecyl sulfate polyacrylamide gel electrophoresis (SDS-PAGE). Harvested cells were disrupted and supernatant fractions boiled for 5 min. Protein concentration was determined using a Bio-Rad Protein Assay Kit (Bio-Rad Laboratories), as described in the manufacturer’s manual. Protein lysate (30 µg) was subjected to 8–10% SDS-PAGE and electrophoretically transferred to a polyvinylidene fluoride membrane (GE Healthcare, Chicago, IL, USA). After blotting, the membrane was incubated with a specific primary antibody at 4 °C overnight. Following hybridization with the secondary antibody, protein bands were visualized using an Amersham enhanced chemiluminescence (ECL) plus western blotting detection system (Piscataway, NJ, USA). MMP-2, GAPDH or β-actin was used as loading control according to previous study [26,27].

### 2.6. Reverse Transcription-Polymerase Chain Reaction (RT-PCR)

For total RNA extraction, HaCaT were treated with PGG for 1 h before being exposed to UVB (30 mJ/cm^2^). After 4 h, total RNA was prepared using a RNeasy kit from Qiagen (Valencia, CA, USA). Purified total RNA 1 mg was reversed-transcribed with oligo-dT primers using a PrimeScript First Strand cDNA Synthesis Kit from TaKaRa Bio Inc. (Kusatsu, Shiga Prefecture, Japan). Amplification consisted of 20 or 25 cycles: denaturation at 95 °C for 45 s, annealing at 50 °C (Glyceraldehyde 3-phosphate dehydrogenase, GAPDH) or 52 °C (MMP-1) for 45 s, and extension at 72 °C for 45 s followed by a final 10 min extension at 72 °C. PCR was performed using a C1000 Touch™ Thermal Cycler (Bio-Rad Laboratories) in a reaction mixture (50 mL) containing EmeraldAmp GT PCR master mix (TaKaRa), 60 ng of cDNA, and 0.2 mM of each primer (Macrogen, Seoul, Korea). cDNA was probed with the following primers as previously reported (Reference); GAPDH (GenBank accession number NM_002046, 635 bp) forward 5’-ATGTTCCAATATGATTCCAC-3’, reverse 5’-TCATCATAT TTGGCAGGTTT-3’; MMP-1 (NM_002421.3, 530 bp) forward 5’-AATACCTGGAAAAATACTAC-3’, reverse 5’-TAAGTTGTACTCTCTGAAAT-3’; GAPDH was used as a control and variations of mRNA concentration were normalized to GAPDH using Image J program from NIH (Bethesda, MD, USA).

### 2.7. In Vitro PAK1 and JNK1 Kinase Assays

In vitro ADP-Glo^TM^ kinase assays were performed in accordance with the instructions provided by Promega (Madison, WI, USA). Briefly, each reaction contained 1× kinase reaction buffer, ADP-Glo^TM^ reagent and detection buffer. For PAK1 or JNK1, 0.2 µg/µL of the inactive PAK1 substrate peptide or 0.4 µM of the inactive ATF substrate peptide was included. In a 96-well plate, 10 µL PAK1 or JNK1 kinase were added to 5 µL PGG and 1× kinase buffer (as a control), and incubated for 15 min at room temperature. After PAK1 or JNK1 kinases were allowed to react with PGG from the reaction mixture, 10 µL ATP-PAK1 kinase or –ATF substrate peptide mixture was added and incubated at 30 °C for 30 min. After the kinase reaction, 25 μL ADP-Glo™ reagent was added to all wells in assay plates and incubated at room temperature for 40 min. Next, 50 μL kinase detection reagent were added to all wells in assay plates and incubated at room temperature for 60 min. The luminescence to integration time of 0.5 sec was measured using a Glomax 96 microplate luminometer (Promega). Each experiment was repeated three times.

### 2.8. In Vitro PAK1 and JNK1 Binding Assays

Recombinant PAK1 or JNK1 (50 ng), or a HaCaT cellular supernatant fraction (1000 μg) was incubated with PGG-Sepharose 4B beads (or Sepharose 4B as a control) (100 μL, 50% slurry) in reaction buffer. After incubation with gentle rocking overnight at 4 °C, beads were washed five times with washing buffer, and proteins bound to the beads were analyzed by immunoblotting. The intensity of the protein band was expressed as relative value to the input control.

### 2.9. ATP and PGG Competition Assays

Briefly, active PAK1 or JNK1 (0.2 μg) was incubated with ATP (10 or 100 μM) in reaction buffer (see “In vitro binding assay” above) for 12 h at 4 °C. PGG-Sepharose 4B (100 μL) or Sepharose 4B (100 μL) was added to a final volume of 500 μL for 12 h. Samples were then washed and proteins detected by Western blot as described above. The intensity of the protein band was expressed as value relative to the input control.

### 2.10. Experimental Animals

SKH-1 hairless mice (5–week–old female) were purchased from Central Laboratory Animal, Inc. (Seoul, Korea). Mice were acclimated for one week prior to experiments and had free access to food and water. Animals were housed under climate-controlled conditions (24 °C at 50% humidity) with a 12 h light/dark cycle. All experiments with mice were performed in accordance with the regulations and approval of the Institutional Animal Care and Use Committee at Kyungpook National University (KNU 2013-49).

### 2.11. UV Irradiation of Hairless Mice

UVB irradiation was performed using a Bio-Link Cross linker (Vilber Lourmat), emitting wavelengths with peak emission at 312 nm. SKH-1 hairless mice were randomly divided into four groups (𝑛 = 5 per group): an untreated control group, an UVB-treated group, and groups treated with UVB plus a low or high dose of PGG (4 or 20 mg/kg). All mice received PGG or vehicle (2% dimethylsulfoxide [DMSO]/PBS) orally 1 h before UVB irradiation. The minimal erythema dose (MED) on mouse dorsal skin was measured initially and 25 mJ/cm^2^ was defined as one MED. The dorsal skin of hairless mice was exposed to UVB 3 times a week and the irradiation dose was increased weekly by 1 MED to 7 MED, and then maintained at 7.2 MED (180 mJ/cm^2^) until 10 weeks.

### 2.12. Preparation of Skin Lysates

Mice were sacrificed by cervical dislocation following their final UVB exposure, and the dorsal skin region of each mouse was excised. After fat removal, the skin was immediately pulverized with liquid nitrogen using a mortar and pestle. Pulverized skin was homogenized on ice with a bullet blender^®^ (Next advance, Troy, NY, USA), and skin lysates were centrifuged at 21,200× *g* for 20 min at 4 °C. Protein content in supernatant fractions was then determined using Bio-Rad Protein Assay Kit (Bio-Rad). Each protein level was analyzed by western blotting as described above.

### 2.13. Generation of Replicas and Image Analysis

After eight weeks, skin samples from each mouse were assessed visually and using a replica assay. Replicas of mouse dorsal skin were prepared using SILFLO Resin (Cuderm, Dallas, TX, USA) and analyzed at the Oriental Medicine Industry Support Center (Daegu, Korea). Parameters used for the assessment of skin wrinkles were average length and depth of wrinkles.

### 2.14. Measurement of Transepidermal Water Loss (TEWL)

TEWL was measured using a Tewameter TM300 (Courage+Khazaka Electronic GmbH, Cologne, Germany) according to the manufacturer’s instructions.

### 2.15. Histological Examination

Samples from the dorsal skin of each treatment group were fixed in 10% neutral-buffered formalin. Paraffin-embedded back skin samples were sectioned and stained with hematoxylin and eosin. Epidermal thickness was measured under light microscopy (Olympus, Tokyo, Japan).

### 2.16. Immunohistochemical Staining

Following deparaffinixation and hydration, sections were quenched with 0.3% hydrogen peroxide, preblocked with 5% normal horse serum for 30 min, and then incubated with the specified primary antibodies overnight. Primary antibodies were diluted in 0.1 M PBS. After blocking, sections were washed three times with 0.01 M PBS and incubated with the appropriate secondary antibodies (1:200) for 2.5 h. Sections were then incubated with ABC solution. Antigens were detected using 3’-diaminobensidine tetrahydrochloride hydrate (DAB) solution. The stained sections were evaluated after dehydration and mounting.

### 2.17. Statistical Analysis

Data are expressed as the means ± standard deviation (S.D.) or standard error (S.E.). Values not sharing common letter on bar indicate statistically significant difference from each other (*p* < 0.05). Statistical significance was determined using one-way analysis of variance (ANOVA). All analyses were performed using SPSS Statistics software (SPSS, Inc., Endicott, NY, USA).

## 3. Results

### 3.1. PGG Inhibits UVB-Induced MMP-1 Expression in HaCaT Cells

Since elevated MMP-1 levels are closely associated with photoaging [28], we investigated the effects of PGG on UVB-induced MMP-1 expression in HaCaT cells. It has been well-reported that UVB strongly induced MMP-1 mRNA and protein level and subsequently MMP-1 was secreted into culture media in cell model systems [26,27,29,30]. In contrast, changes in MMP-2 mRNA and protein levels by UVB irradiation were not observed or very slightly increased in human keratinocytes [27,28]. Therefore, MMP-2 was used as loading control. PGG significantly inhibited UVB-induced MMP-1 secretion into culture media of HaCaT cells at non-toxic concentrations (Figure 1B and Figure S1). Consistently, PGG suppressed level of MMP-1 protein and mRNA induced by UVB (Figure 1C,D, respectively). Furthermore, we examined whether PGG exerts an inhibitory effect on in vitro MMP-1 activity. However, PGG did not show significant inhibitory effect on MMP-1 activity up to 20 μM (Figure 1E). These results indicate that PGG is effective at inhibiting UVB-induced MMP-1 expression and secretion in HaCaT cells, but not in vitro MMP-1 activity.

### 3.2. PGG Suppresses UVB-Induced RAF-MEK-ERK-p90^RSK^, MKK3/6-p38-MSK1, and c-Jun, but not MKK4/7-JNK in HaCaT Cells

**Figure 2:**
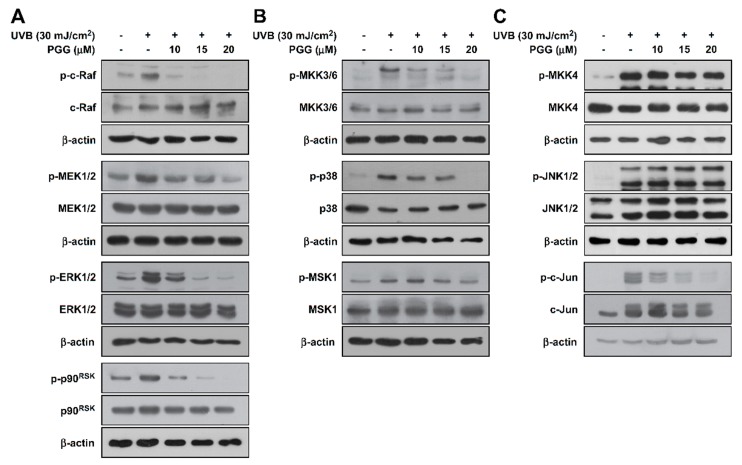
Effect of PGG on UVB-induced MAPK signaling in HaCaT cells. (**A**) Phosphorylation of ERK signaling (RAF, MEK, ERK, p90^RSK^), (**B**) phosphorylation of p38 signaling (MKK3/6, p38, MSK1) and (**C**) phosphorylation of JNK signaling (MKK4, JNK, c-Jun). Cell pretreated with PGG (10, 15 and 20 μM) for 1 h were harvested 15 min after exposure to UVB (30 mJ/cm^2^). Western blot analysis was performed to determine levels of phosphorylated and total RAF, MEK, ERK, p90^RSK^, MKK3/6, p38, MSK1, MKK4, JNK and c-Jun proteins were determined by western blot analysis. β-actin was used as loading control. Data are representative of three independent experiments that yielded similar results.

Previous studies have shown that the MAPK signaling pathway is clearly involved in UVB-induced MMP-1 expression in HaCaT cells [12,31]. Thus, we investigated the effect of PGG on UVB-induced MAPK phosphorylation in HaCaT cells. PGG suppressed UVB-induced phosphorylation of the ERK (RAF-MEK-ERK-p90^RSK^) (Figure 2A and Figure S2A) and p38 signaling pathways (MKK3/6-p38-MSK1) (Figure 2B and Figure S2B) in HaCaT cells at the indicated concentrations. PGG did not inhibit UVB-induced phosphorylation of MKK4 and JNK, but did inhibit c-Jun (Figure 2C and Figure S2C). Overall, these results confirm that PGG is an effective suppressor of UVB-induced phosphorylation of ERK and p38 signaling pathways, as well as c-Jun.

### 3.3. PAK1 is Involved in UVB-Induced MMP-1 Upregulation by Modulating the Activation of ERK and p38 Pathways

**Figure 3:**
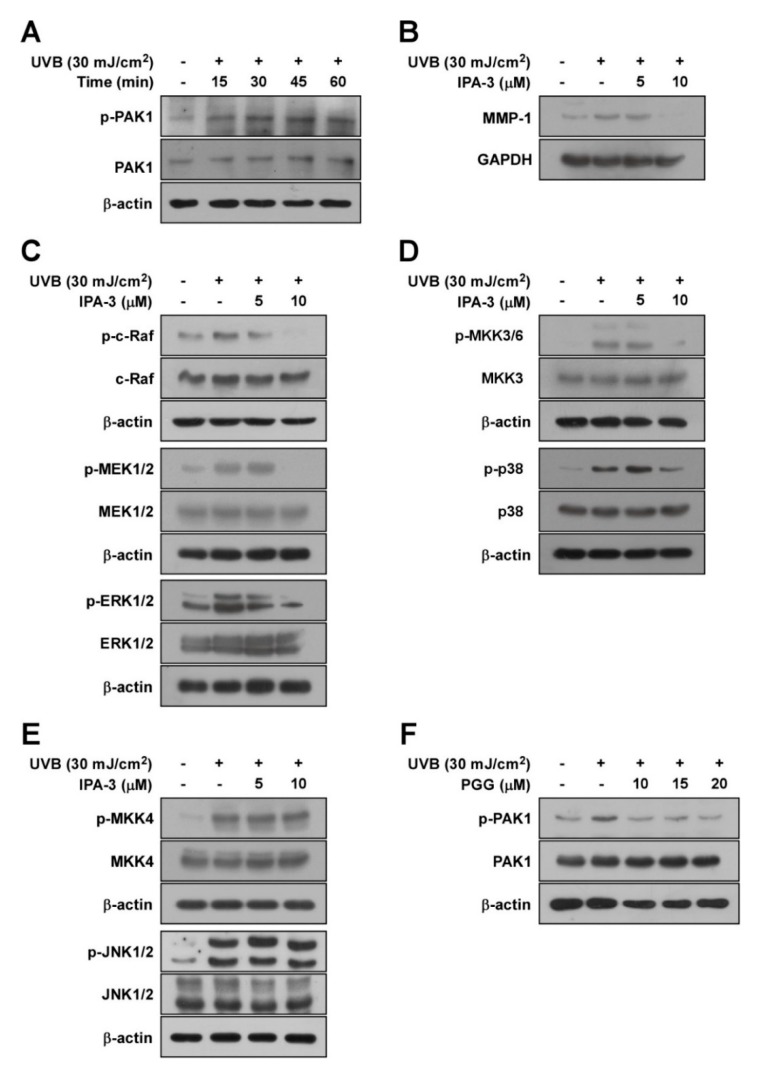
The role of PAK1 on UVB-induced MMP-1 expression in HaCaT cells. (**A**) A time course of UVB-induced phosphorylation of PAK1 in HaCaT cells. After serum-free starvation, cell were irradiated with UVB (30 mJ/cm^2^) at different time points as indicated, whole cell lysates were collected. (**B**) Effect of IPA-3 on UVB-induced MMP-1 expression in HaCaT cells. Cells pretreated with IPA-3 (5 and 10 μM) for 1 h were harvested 4 h after exposure to UVB (30 mJ/cm^2^). (**C**–**E**) Inhibitory effect of IPA-3 on UVB-induced phosphorylation of ERK, p38 and JNK signaling in HaCaT cells. Cells pretreated with IPA-3 (5 and 10 μM) for 1 h were harvested 15 min after exposure to UVB (30 mJ/cm^2^). Cells were treated with IPA-3 (5 and 10 μM) for 1 h before being exposed to UVB (30 mJ/cm^2^) and harvested 15 min later. (**F**) Effect of PGG on UVB-induced phosphorylation of PAK1. Cells were treated with PGG (10, 15 and 20 μM). After 1h, the cells were exposed to UVB (30 mJ/cm^2^) and harvested 15 min later. Western blot analysis was used to determine levels of MMP-1 expression and RAF, MEK, ERK, p90^RSK^, MKK4, JNK, MKK3/6, p38, and PAK1 phosphorylation using specific antibodies against the corresponding phosphorylated and total proteins. β-actin was used as loading control. Data are representative of three independent experiments that yielded similar results.

PAK1 reportedly acts as a regulator of MMP-1 by modulating MAPKs [32]. Therefore, we hypothesized that PAK1 suppression by PGG inhibits UV-induced MMP-1 expression by acting as an upstream kinase of ERK and p38 signaling. First, we examined phosphorylation levels of PAK1 following UVB irradiation at the indicated times. PAK1 phosphorylation was increased by UVB within 45 min, but slightly decreased after 60 min (Figure 3A and Figure S3A). To confirm the involvement of PAK1 in UVB-stimulated MMP-1 induction, we used IPA-3, an effective PAK1 inhibitor. IPA-3 is known to block or stabilize PAK homodimers in an autoinhibitory state via covalent binding to the PID sequence of PAKs [33]. Treatment with IPA-3 suppressed UVB-induced MMP-1 expression (Figure 3B and Figure S3B). Moreover, IPA-3 inhibited UVB-induced phosphorylation of RAF/MEK/ERK (Figure 3C and Figure S3C) and MKK3/6-p38-MSK1 (Figure 3D and Figure S3D), but not MKK4-JNK (Figure 3E and Figure S3E). These results support that PAK1 is required for UVB-induced MMP-1 upregulation by modulating ERK and p38 pathways in HaCaT cells. Consistently, PGG reduced UVB-induced phosphorylation of PAK1 (Figure 3F and Figure S3F), revealing that inactivation of PAK1 is attributed to the inhibitory mechanism of MMP-1 expression by PGG. Accumulating data suggest that PAK1 plays a significant role in regulating MMP-1 expression as a possible upstream kinase of ERK and p38 pathways in HaCaT cells.

### 3.4. PGG Inhibits PAK1 and JNK1 Kinase Activities In Vitro

**Figure 4:**
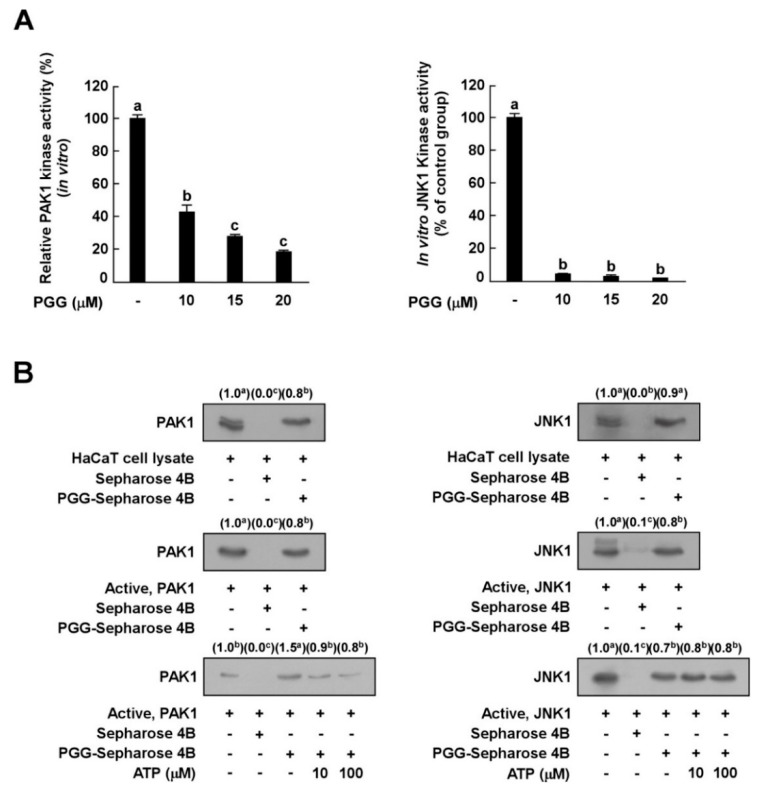
Effect of PGG on PAK1 and JNK1 kinase activities. (**A**) Kinase activities are expressed as the percentage of relative inhibition to untreated control PAK1 or JNK1 activities. Data are presented as the mean ± S.D., as determined from three independent experiments. (**B**) Binding of PGG with PAK1 or JNK1. Western blot analysis using antibodies against PAK1 or JNK1 was executed to confirme the PAK1- or JNK1-PGG binding in vitro (**B**, upper panels); first lane (input control), PAK1 or JNK1 protein standard; second lane (negative control), PAK1 and JNK1 with Sepharose 4B; third lane, PAK1 and JNK1 with PGG-Sepharose 4B beads. Middle panels; first lane (input control), whole cell lysate from HaCaT cells; second land (negative control), lysate from HaCaT cells precipitated with Sepharose 4B beads; third lane, whole cell lysate from HaCaT cells precipitated with PGG-Sepharose 4B affinity beads. Lower panels; first lane (input control), active PAK1 or JNK1; second lane (negative control), PAK1 and JNK1 with Sepharose 4B beads; third lane (positive control), PAK1 and JNK1 with PGG-Sepharose 4B beads; fourth and fifth lanes, increasing concentrations of ATP suppress the binding of PGG to PAK1, but not JNK1. Data are representative of three independent experiments that yielded similar results. The values in parentheses represent the means of the relative intensity to the input control (first lane), and values that do not share common letter (a,b,c) on bar indicate statistically significant difference from each other (*p* < 0.05).

Since PGG inhibited UVB-induced c-Jun phosphorylation, but not JNKs, we hypothesized that PGG would decrease the phosphorylation of c-Jun by inhibiting JNK kinase. Based on the above results, we next examined the effect of PGG on PAK1 or JNK1 kinase activity in vitro to investigate whether PAK1 or JNK1 kinase are molecular targets of PGG in relation to UVB-induced MMP-1 expression. In vitro kinase assay data showed that PGG strongly inhibited PAK1 and JNK1 kinase activity (Figure 4A), indicating that PAK1 and JNK1 are potential molecular targets of PGG.

### 3.5. PGG Binds with PAK1 in an ATP-Competitive Manner and JNK1 in an ATP-Independent Manner

The results described above indicated that PGG is involved in the suppression of PAK1 and JNK1 activities and subsequent inhibition of downstream signaling pathways. To determine whether PGG directly interacts with PAK1 or JNK1, we performed an in vitro pull-down assay. PAK1 was observed in PGG–Sepharose 4B beads (Figure 4B, left upper panel, lane 3), but not Sepharose-4B-only beads (Figure 4B, left upper panel, lane 2). PAK1 protein was loaded as a control (Figure 4B, left upper panel, lane 1). We also observed binding of PGG and PAK1 in HaCaT cell lysates (Figure 4B, left middle panel). PGG competed with ATP to bind with PAK1 (Figure 4B, left lower panel). In addition, PGG directly interacted with JNK1 in vitro (Figure 4B, right upper or middle panels). PGG did not compete with ATP for binding to JNK1 (Figure 4B, right lower panel). Collectively, these results indicate that PGG inhibits PAK1 activity in an ATP-competitive manner and JNK1 kinase activity in an ATP-noncompetitive manner.

### 3.6. PGG Inhibits UVB-Induced Photoaging Symptoms In Vivo

**Figure 5:**
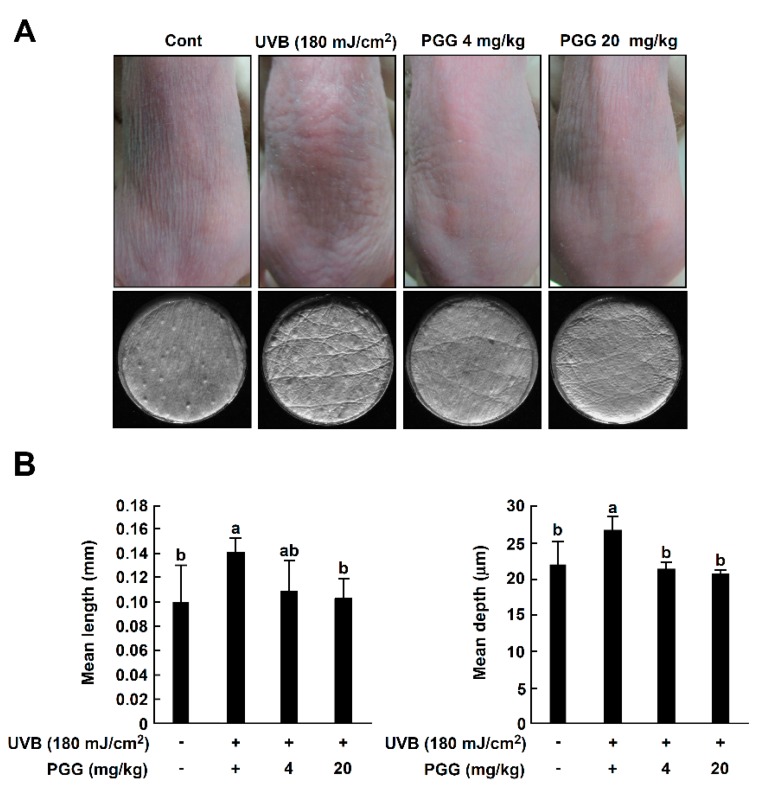

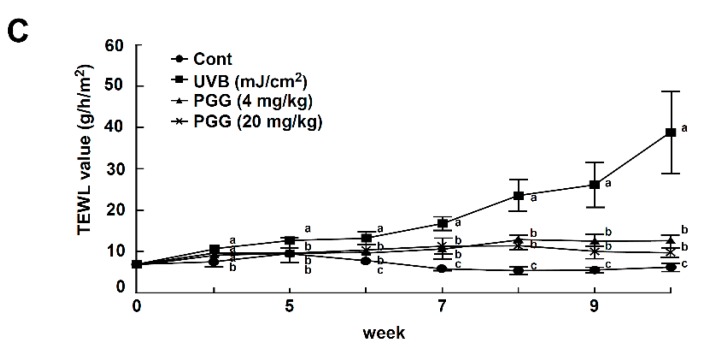
Effect of PGG on UVB-induced photoaging symptoms in SKH-1 hairless mice. (**A**) Anti-photoaging effect of PGG on visual wrinkle formation by UVB irradiation in SKH-1 hairless mice. (**B**) Analysis of replicas for the effect of PGG on UVB-induced mean length and depth of skin wrinkles. (**C**) Changes in TEWL of SKH-1 hairless mice dorsal skin during the experimental period. Results are shown as the means ± S.D. (*n* = 5). Values that do not share common letter (a,b,c) on bar indicate statistically significant difference from each other (*p* < 0.05).

To investigate the effect of PGG on UVB-induced photoaging symptoms in vivo, we measured wrinkle formation and transepidermal water loss (TEWL) using SKH-1 hairless mice [4,34]. Dorsal skin was exposed to UVB (25–180 mJ/cm^2^) 1 h following PGG oral administration, three times per week for a total of 10 weeks. Representative photographs of mice at the conclusion of the treatment duration showed that PGG inhibited UVB-induced wrinkle formation (Figure 5A, upper pictures). To further evaluate the inhibitory activity of PGG on wrinkles, replicas of mouse dorsal skin (Figure 5A, lower pictures) were analyzed and quantified using an image analysis system [35]. In the UVB-treated group, longer and deeper coarse wrinkles were observed across the dorsal skin, and the mean length and depth of wrinkles was significantly increased compared to that of the control group (Figure 5B).

Treatment with PGG (4 or 20 mg/kg) improved the mean length and depth of wrinkles compared to the UVB group (Figure 5B). According to a previous study, UVB exposure causes skin dehydration and TEWL of the skin, leading to the visible appearance of dryness and wrinkles [36,37]. Thus, we confirmed the level of TEWL on mouse dorsal skin following 10 weeks of UVB exposure. TEWL levels in the UVB irradiated group were increased in a time-dependent manner, and PGG (4 or 20 mg/kg) suppressed UVB-induced TEWL levels similar to that of the control group (Figure 5C). There was no significant difference in PGG between the 4 and 20 mg/kg groups. Our results indicate that oral administration of PGG attenuated photoaging symptoms in SKH-1 hairless mice.

### 3.7. PGG Suppresses UVB-Induced Epidermal Thickening In Vivo

**Figure 6:**
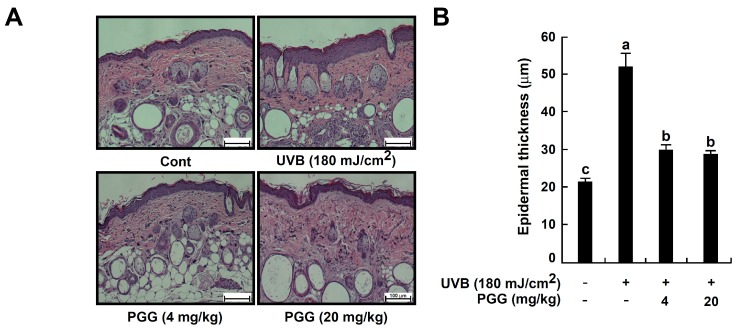

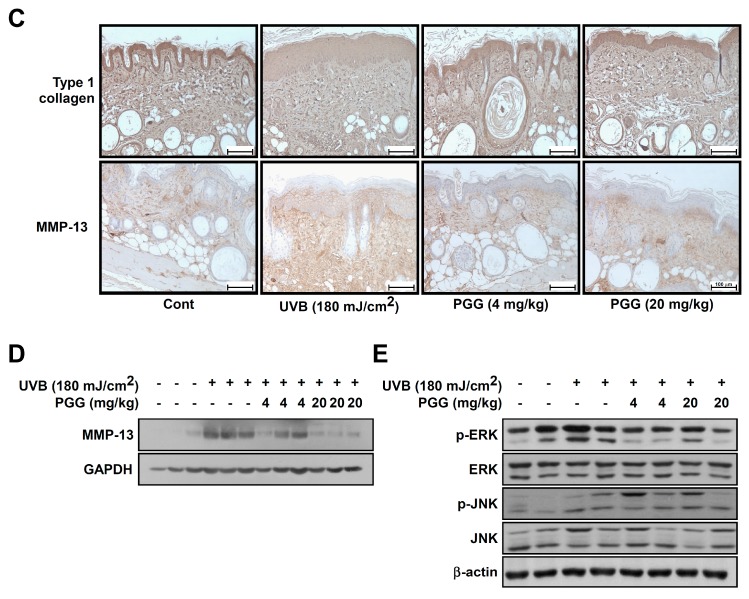
Effect of PGG on histological and molecular properties in mouse dorsal skin following UVB irradiation. (**A**) Histological evaluation by hematoxylin and eosin-staining of UVB-irradiated mouse skin. Images are representative of five tissue samples. (**B**) Effect of PGG on UVB-induced epidermal thickening. The epidermal thickness was measured using image in Figure 6A. Bars represent the mean thickness (μm) of the epidermis from five animals. Results are shown as the means ± standard error (S.E.) (*n* = 5). Values that do not share common letter (a,b,c) on bar indicate statistically significant difference from each other (*p* < 0.05). (**C**,**D**) Effect of PGG on UVB-induced type 1 collagen degradation and MMP-13 expression on mouse dorsal skin. Immunohistochemstry and western blot analysis were performed as described in ’Materials and Methods’ using specific antibodies for each protein. (**E**) Effect of PGG on UVB-induced MAPK signaling pathway on mouse dorsal skin. Protein was extracted from mouse dorsal skin as described in ‘Materials and Methods’. Western blot analysis was performed to determine levels of phosphorylated and total ERK and JNK proteins.

Epidermal thickness is used as a quantitative parameter to assess skin photoaging [26,38]. Therefore, we evaluated the effect of PGG on UVB-induced epidermal thickening (Figure 6). Hematoxylin and eosin staining demonstrated that UVB irradiation significantly increased epidermal thickness. The UVB-treated group (51.79 ± 3.44 μm) exhibited an almost 2.5-fold increase in epidermal thickness compared to the untreated control group (21.36 ± 0.76 μm). PGG (4 or 20 mg/kg) significantly reversed the increase in epidermal thickening by 29.89 ± 1.17 and 28.70 ± 0.56 μm, respectively (Figure 6A,B).

### 3.8. PGG Increases UVB-Induced Type 1 Collagen Degradation and Inhibits UVB-Induced MMP-13 Expression In Vivo

To examine the inhibitory effect of PGG on UVB-induced photoaging, we measured the levels of type 1 collagen using immunohistochemical staining. UVB exposure causes collagen degradation, and this process contributes to increases in the level of wrinkles [39]. Levels of collagen fibers were reduced in the upper dermis of the UVB group compared with the control groups, as observed by weaker staining of the irradiated skin. PGG oral administration (4 or 20 mg/kg) prevented this reduction (Figure 6C, upper picture), suggesting that PGG inhibited UVB-induced degradation of type 1 collagen in mouse dorsal skin.

Matrix-metalloproteinase (MMP) expression by UV exposure is the major cause of collagen destruction [12]. Mouse MMP-13, a rodent interstitial collagenase, has structural and functional homology very close to human MMP-13, it can replace human MMP-1 in murine skin [40,41,42]. It also can cleave collagen type I, II, III or X in a native triple helical domain like MMP-1 and -8 [4]. In addition, it has been shown to be induced by c-Jun and c-Fos activity in UVB-irradiated hairless mice [43]. Therefore, we determined whether the photoprotective effect of PGG was associated with a change in UVB-induced MMP-13 expression using immunohistochemical staining (Figure 6C, lower pictures) and western blot analysis (Figure 6D and Figure S3A). PGG inhibited UVB-induced MMP-13 expression in mouse skin (Figure 6C, lower pictures). Additionally, MMP-13 expression was increased 8.4-fold in UVB groups of dorsal skin compared to the control group. Furthermore, UVB-induced MMP-13 expression was noticeably inhibited by PGG oral administration (4 or 20 mg/kg) (Figure 6D and Figure S4A). PGG treatment attenuated UVB-induced MMP-13 expression 5.2- and 2.9-fold, respectively. Furthermore, our western blot data showed that PGG also reduced UVB-induced phosphorylation of ERK, but not JNK in vivo (Figure 6E and Figure S4B). These results were consistent with western blot result of HaCaT cells. Therefore, we deduced that anti-photoaging mechanism of PGG in vivo would be same with that of in vitro.

## 4. Discussion

Skin aging can be divided into intrinsic and extrinsic aging (photoaging) [4]. Environmental factors including UV irradiation are known to cause skin aging [12]. UV irradiation, particularly UVB, induces the expression of MMPs, which are involved in the degradation of extracellular matrix components in human and mouse skin [1]. MMP-1 is an interstitial collagenase, and inhibition of MMP-1 overexpression induced by UVB could represent a primary mechanism to prevent photoaging. Previous studies have suggested that natural phytochemicals exhibit an inhibitory effect on MMP-1 expression by targeting and suppressing signaling pathways involved in skin aging [4,37,39,44]. PGG is known to exhibit multiple biological activities, including chemopreventive effects [18,19,20,21]. In particular, a recently previous study has proposed photoprotective effect of PGG based on the anti-inflammatory effect with antioxidant activity in UVB-irradiated human dermal fibroblasts [23]. Based on the study, PGG could be a potential candidate for protecting against UVB-induced skin damage. However, the previous data were not sufficient to explain the effect of PGG on skin aging associated with skin matrix changes such as wrinkling. In the present study, we investigated the anti-photoaging effects of PGG in HaCaT cells and SKH-1 hairless mice, and its molecular mechanisms. Our results show that PGG suppresses UVB-induced MMP-1 secretion into cell culture media and MMP-1 expression in HaCaT cells. However, PGG had no effect on in vitro MMP-1 activity up to 20 μM. These results suggest that PGG could inhibit UVB-induced collagen degradation by reducing the MMP-1 amount itself that can act collagen, rather than regulating its enzymatic activity.

The MAPK signal transduction pathways are required for MMP expression regulation [45,46]. Accumulating evidence suggests that the MAPK family is involved in MMP upregulation, resulting in photodamaged skin. Our results demonstrate that PGG inhibited UVB-induced phosphorylation of ERK and p38 signal pathways, as well as c-Jun in HaCaT cells. Previous studies showed that PAK1 is involved in several cell signaling pathways. Activation of PAK1 and its downstream signaling pathways, such as mitogen-activated protein kinases (MAPKs) and nuclear factor-κB (NF-κB), are important in carcinogenesis [47]. Therefore, we examined whether PAK1 is related to UVB-induced MMP-1 expression in HaCaT cells as an upstream regulator of ERK and p38 signal pathways. We found that UVB irradiation induced a maximal phosphorylation level of PAK1 after 15 min in HaCaT cells. The use of IPA-3, a PAK1 inhibitor, confirmed that PAK1 is involved in UVB-induced MMP-1 expression and regulation by acting as an upstream regulator of ERK and p38 signal pathways, but not the JNK pathway. In addition, PGG inhibited the phosphorylation of PAK1. Based on these results, we hypothesized that PGG suppresses MMP-1 expression by directly inhibiting the kinase activities of PAK1 and JNK1. In vitro kinase assay results indicated that PGG significantly inhibits PAK1 and JNK1 activities. Furthermore, PGG directly binds with PAK1 in an ATP-competitive manner and JNK1 in an ATP-noncompetitive manner. Therefore, PGG may contribute to its anti-photoaging activity by inhibiting PAK1 and JNK1. The study that reported the photoprotective effect of PGG on human skin cells showed that PGG inhibits the activation of UVB-induced MAPK and NF-kB, but did not fully explain the mechanism and molecular targets [25]. Although we have identified molecular mechanisms in human epidermal keratinocytes (HaCaT cells), our results are consistent with the previous report that PGG inhibits UVB-induced MAPK in human dermal fibroblasts. Furthermore, PAK1, the potential target of PGG identified in our study, may be the upstream mediator of NF-κB as well as MAPK.

Additionally, we confirmed the effect of chronic UVB exposure on skin aging symptoms in a SKH-1 hairless mouse model, which has been widely used for photo-damage studies [4]. We used only females as in vivo model, but previous studies have shown that the photoaging model is not subject to estrous cycles [48,49], therefore, our results could provide information independent of the phase of the estrous cycle. We determined that oral administration of PGG suppressed chronic UVB-induced wrinkle formation in mouse dorsal skin. Skin replica analysis of PGG for measuring the inhibitory activity on photoaging was consistent with our macroscopic observational data. In addition, repeated UV exposure may damage skin barrier function and result in skin photoaging. The level of TEWL was increased in 10 week UVB-treated skin compared with control skin, and treatment with PGG at 4 and 20 mg/kg suppressed the levels of TEWL.

Histological studies have shown that photoaged skin is associated with increased epidermal thickness and changes in the organization of connective tissue [26,50]. Similarly, we found that chronic UVB induced epidermal thickening in mouse skin, while PGG inhibited the increase in epidermal thickness. UVB-induced thickening of epidermis is the result of hyperplasia by repeated repeated cell death and cell proliferation of epidermal cells [51]. It belongs to the disruption of the skin barrier that may be associated with loss of moisture in the skin. Our in vivo data indeed showed that UVB irradiation up-regulated TEWL in mouse dosal, which was alleviated by PGG administration. Ultraviolet irradiation causes major alternations in skin tissues and results in the degradation of collagen, a primary structural component of the extracellular matrix [52]. Levels of type 1 collagen fiber were decreased in the dermis in UVB groups compared to the control group. In contrast, UVB inhibited type 1 collagen degradation in all PGG-treated groups. Subsequently, we observed that PGG-treated groups showed decreased MMP-13 expression compared with UVB-treated groups. Consistent with our previous in vitro observed, these results suggest that PGG suppressed photoaging following chronic UVB exposure. Our statistical analysis of the in vivo data showed that PGG oral administration only exhibited a concentration-dependent effect on wrinkle length and MMP-13 expression in mouse dorsal skin. In contrast, differently treated concentrations of PGG showed no significantly difference in wrinkle depth and epidermal thickness. The reason why two concentration treatments showed no difference would be speculated that one source of the extensive variability in drug concentrations, and consequently, response, within each group can be attributed to differences in drug absorption, distribution and metabolism [53]. It has been well-established that all these factors are very important factors in determining of critical pharmacological properties of a drug, including the efficacy, side effects, and duration of action of the drug [53,54]. Because PGG treatment was performed with oral ingestion, we hypothesize that pharmacokinetic tolerance may be occurred through enzyme induction which is partly responsible for the phenomenon of tolerance. One of reason why pharmacokinetic tolerance occurs may be due to a decreased quantity and effect of the PGG reaching the skin caused by an increase in induction of the enzymes depredating the drug such as CYP450 enzymes. However, there are also other several mechanisms leading to tolerance and exact experimental evidences should be performed to support our hypothesis. Thus, further studies related with drug metabolism and genome analysis in mice are needed to elucidate detailed mechanism in the future.

## 5. Conclusions

**Figure 7:**
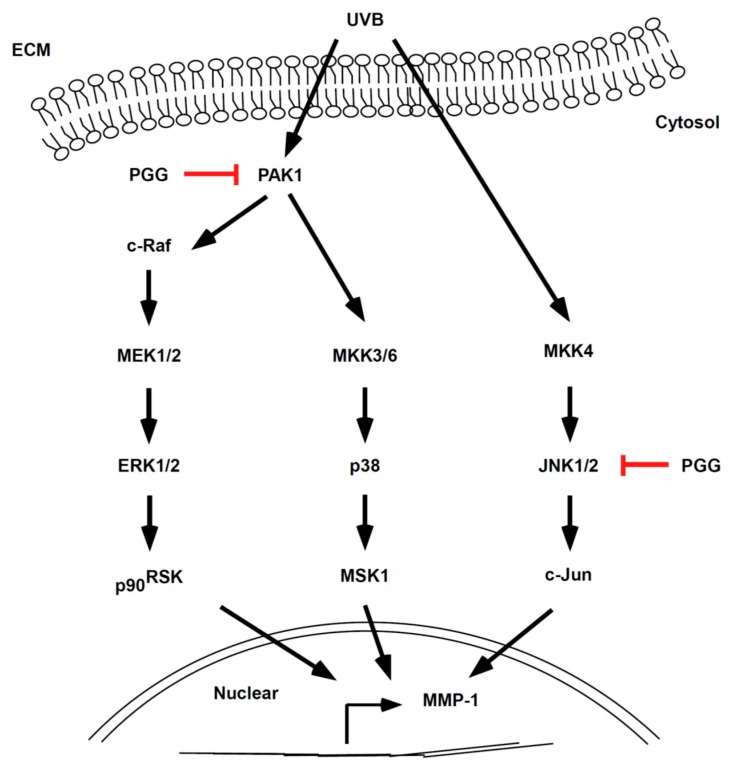
Simplified depiction of the proposed anti-photoaging mechanism of PGG.

In conclusion, PGG inhibited UVB-induced photoaging both in vitro and in vivo. Our results demonstrate that PAK1 and JNK1 kinases represent potential molecular targets of PGG for the inhibition of UVB-induced MMP-1 expression (Figure 7). Therefore, PGG may serve as a useful agent for the prevention of UVB-induced skin photoaging.

## Supplementary Materials

The following are available online at https://www.mdpi.com/2076-3921/8/11/561/s1, Figure S1: Cytotoxicity of PGG on HaCaT cells, Figure S2: Effect of PGG on UVB-induced MAPK signaling in HaCaT cells, Figure S3: The role of PAK1 on UVB-induced MMP-1 expression in HaCaT cells, Figure S4: Effect of PGG on UVB-induced MMP-13 expression and MAPK signaling pathway in vivo.
