# Supplementary material for: Penta-1,2,3,4,6-O-Galloyl-β-d-Glucose Inhibits UVB-Induced Photoaging by Targeting PAK1 and JNK1
Source: Antioxidants (Basel). 2019 Nov 15;8(11):561. doi: 10.3390/antiox8110561 (PMC6912523; doi:10.3390/antiox8110561)
Supplement: Supplementary file 1 [file antioxidants-08-00561-s001.pdf]

antioxidants

MDPI

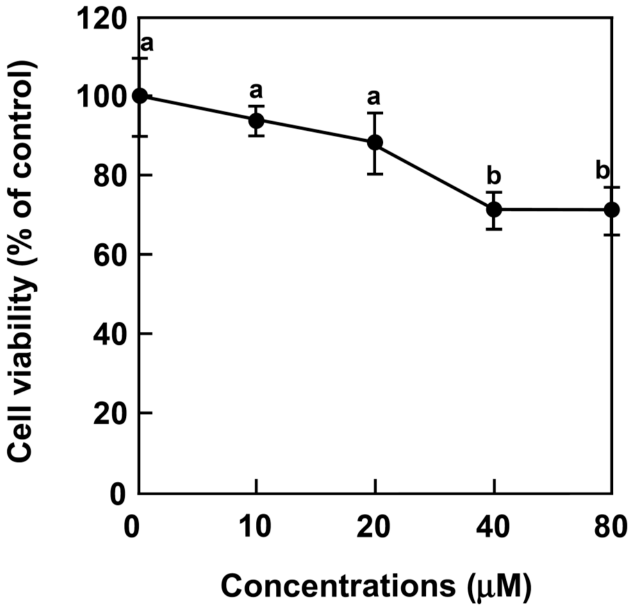

Figure S1. Cytotoxicity of PGG on HaCaT cells. PGG at the indicated concentrations were treated in HaCaT cells for 24 h. The viability of HaCaT cells was determined by MTT assay as described in 'Materials and Methods'. Data are are means ± S.D. as relative percentage to the untreated control. Values not sharing common letter (a,b) on bar indicate statistically significant difference from each other ($p < 0.05$).

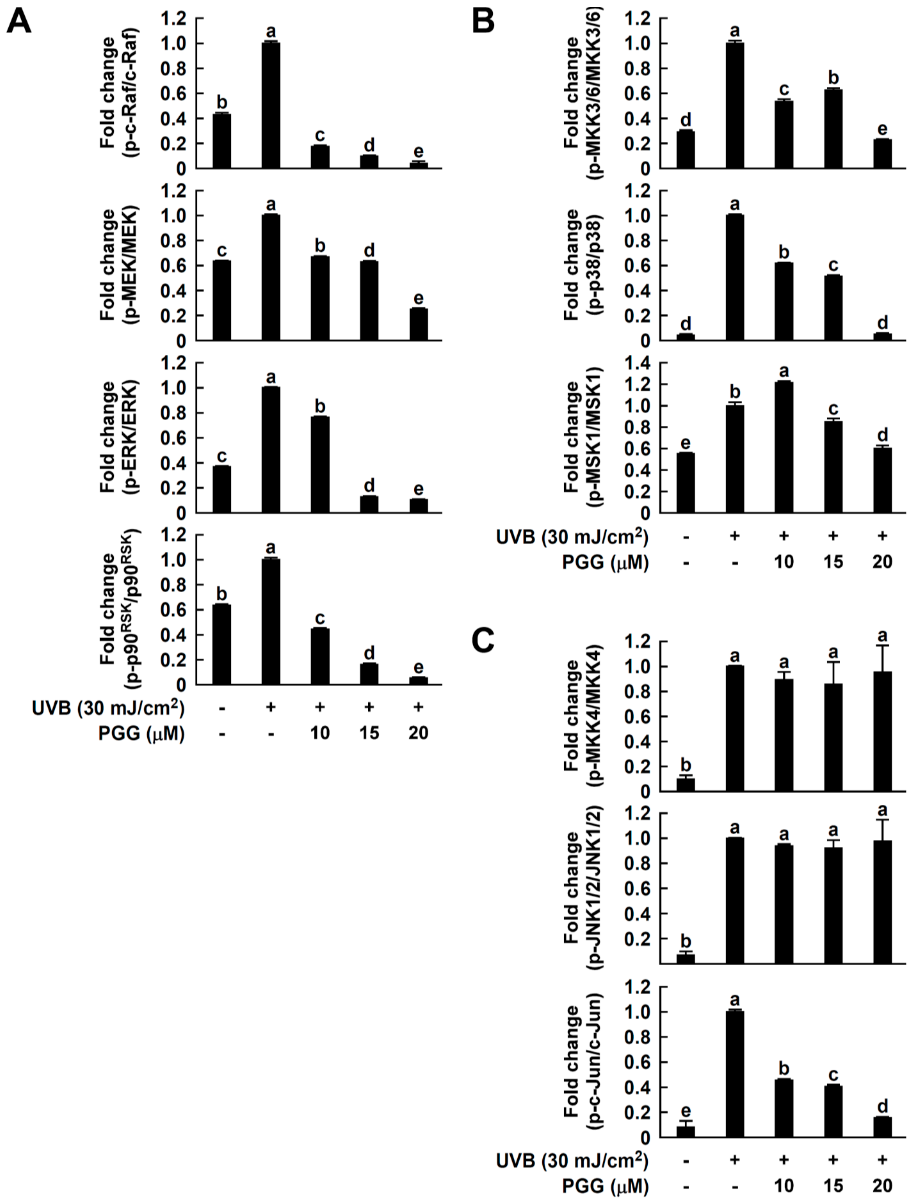

Figure S2. Effect of PGG on UVB-induced MAPK signaling in HaCaT cells. (A) Phosphorylation of ERK signaling (RAF, MEK, ERK, p90RSK), (B) phosphorylation of p38 signaling (MKK3/6, p38, MSK1) and (C) phosphorylation of JNK signaling (MKK4, JNK, c-Jun). Cells were treated with PGG (10, 15 and 20 μM) for 1 h before being exposed to UVB (30 mJ/cm2) and harvested 15 min later. Levels of phosphorylated and total RAF, MEK, ERK, p90RSK, MKK3/6, p38, MSK1, MKK4, JNK and c-Jun proteins were determined by Western blot analysis and quantified using the Image J software. The intensity represents the means ± S.D. of the fold change of phospho/total form ratio relative to the UVB-treated group. Values not sharing common letter (a,b,c,d,e) on bar indicate statistically significant difference from each other ($p < 0.05$).

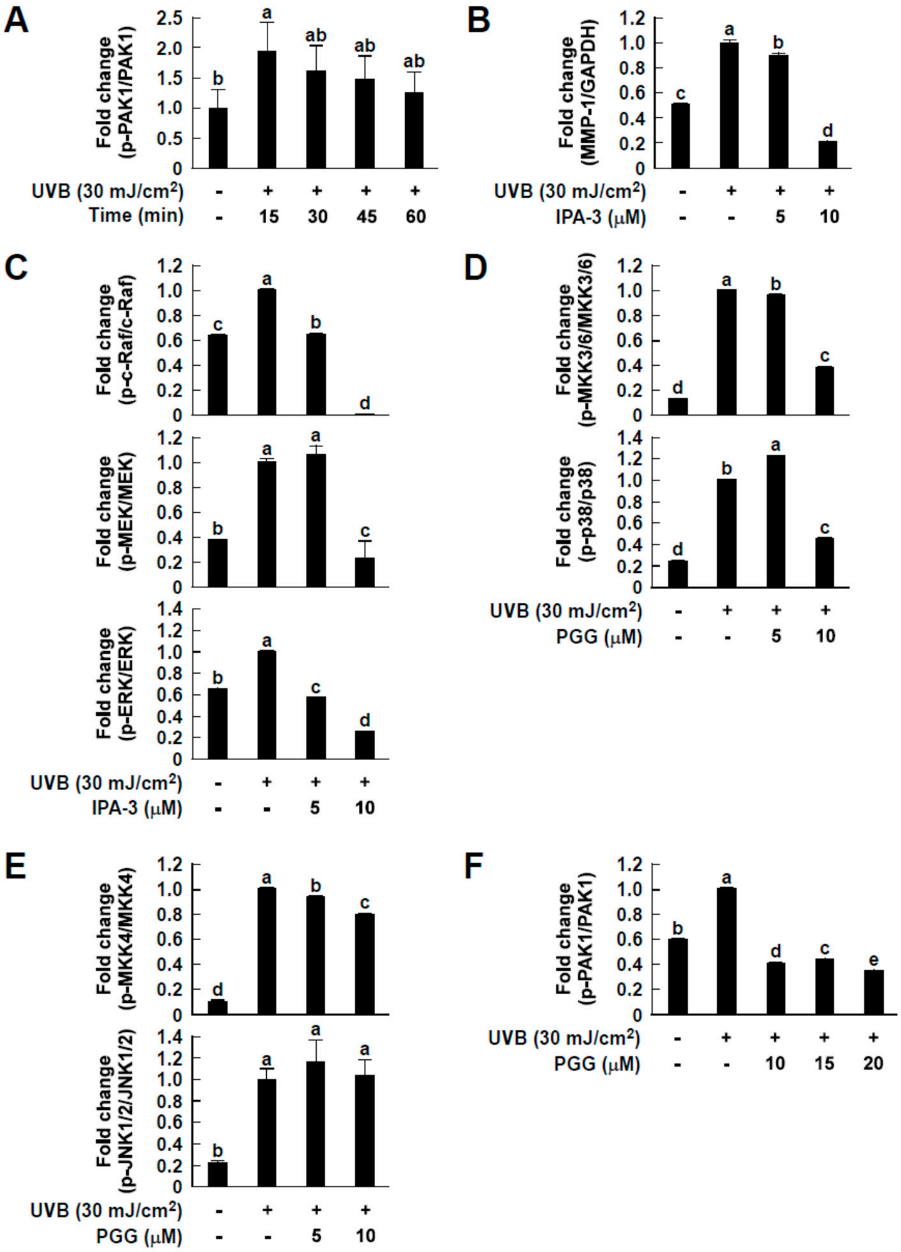

Figure S3. The role of PAK1 on UVB-induced MMP-1 expression in HaCaT cells. (A) A time course of UVB-induced phosphorylation of PAK1 in HaCaT cells. Cells were irradiated with UVB (30 mJ/cm^2) after 24 h of serum-free starvation, whole cell lysates were collected at different time points as indicated. (B) Effect of IPA-3 on UVB-induced MMP-1 expression in HaCaT cells. Cells were treated with IPA-3 (5 and 10 μM) for 1 h before being exposed to UVB (30 mJ/cm^2) and harvested 4 h later. (C, D, E) Inhibitory effect of IPA-3 on UVB-induced phosphorylation of ERK, p38 and JNK signaling in HaCaT cells. Cells were treated with IPA-3 (5 and 10 μM) for 1 h before being exposed to UVB (30 mJ/cm^2) and harvested 15 min later. (F) Effect of PGG on UVB-induced phosphorylation of PAK1. Cells were treated with PGG (10, 15 and 20 μM) for 1 h before being exposed to UVB (30 mJ/cm^2) and

harvested 15 min later. Levels of MMP-1 expression and RAF, MEK, ERK, p90RSK, MKK4, JNK, MKK3/6, p38, and PAK1 phosphorylation were determined by Western blot analysis and quantified using the Image J software. The intensity represents the means ± S.D. of the fold change of phospho/total form ratio relative to the UVB-treated group. Values not sharing common letter (a,b,c,d) on bar indicate statistically significant difference from each other ($p < 0.05$).

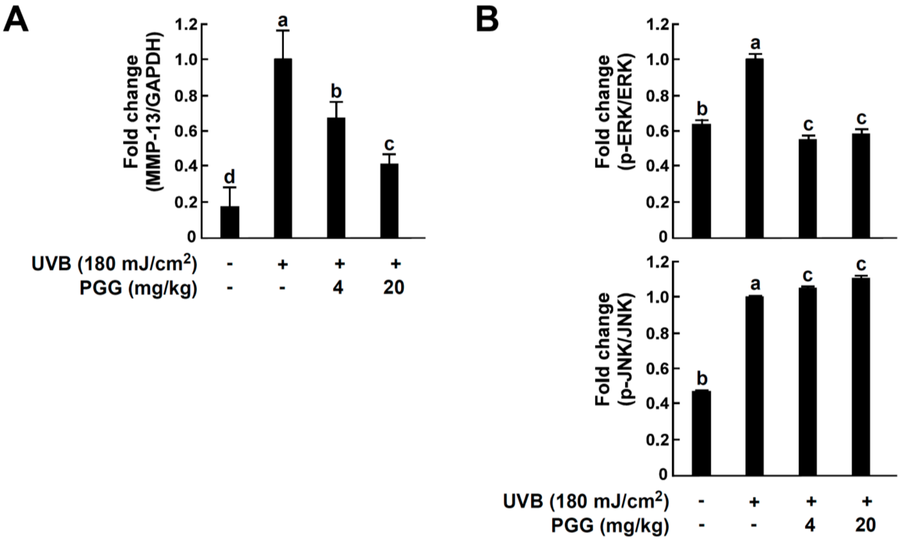

Figure S4. Effect of PGG on UVB-induced MMP-13 expression and MAPK signaling pathway *in vivo*. (A) Effect of PGG on UVB-induced MMP-13 expression on mouse dorsal skin. Protein was extracted from mouse dorsal skin as described in 'Materials and Methods' and MMP-13 expression was determined by Western blot analysis using specific antibodies against MMP-13 protein. (B) Effect of PGG on UVB-induced MAPK signaling pathway on mouse dorsal skin. Protein was extracted from mouse dorsal skin as described in 'Materials and Methods'. Levels of phosphorylated and total ERK and JNK proteins were determined by Western blot analysis.The data were quantified using the Image J software and intensity represents the means ± S.D. of the fold change relative to the UVB-treated group. Values not sharing common letter (a,b,c,d) on bar indicate statistically significant difference from each other ($p < 0.05$).
